# Serum metabolome analysis in hyperthyroid cats before and after radioactive iodine therapy

**DOI:** 10.1371/journal.pone.0305271

**Published:** 2024-06-10

**Authors:** Molly A. Bechtold, Yimei Lin, Meredith L. Miller, Jennifer M. Prieto, Carol E. Frederick, Lucinda L. Bennett, Mark E. Peterson, Kenneth W. Simpson, John P. Loftus

**Affiliations:** 1 Department of Clinical Sciences, College of Veterinary Medicine, Cornell University, Ithaca, New York, United States of America; 2 Animal Endocrine Clinic, New York, New York, United States of America; Taylor’s University - Lakeside Campus: Taylor’s University, MALAYSIA

## Abstract

Hyperthyroidism is the most common feline endocrinopathy. In hyperthyroid humans, untargeted metabolomic analysis identified persistent metabolic derangements despite achieving a euthyroid state. Therefore, we sought to define the metabolome of hyperthyroid cats and identify ongoing metabolic changes after treatment. We prospectively compared privately-owned hyperthyroid cats (n = 7) admitted for radioactive iodine (I-131) treatment and euthyroid privately-owned control (CON) cats (n = 12). Serum samples were collected before (T0), 1-month (T1), and three months after (T3) I-131 therapy for untargeted metabolomic analysis by MS/MS. Hyperthyroid cats (T0) had a distinct metabolic signature with 277 significantly different metabolites than controls (70 increased, 207 decreased). After treatment, 66 (T1 vs. CON) and 64 (T3 vs. CON) metabolite differences persisted. Clustering and data reduction analysis revealed separate clustering of hyperthyroid (T0) and CON cats with intermediate phenotypes after treatment (T1 & T3). Mevalonate/mevalonolactone and creatine phosphate were candidate biomarkers with excellent discrimination between hyperthyroid and healthy cats. We found several metabolic derangements (e.g., decreased carnitine and α-tocopherol) do not entirely resolve after achieving a euthyroid state after treating hyperthyroid cats with I-131. Further investigation is warranted to determine diagnostic and therapeutic implications for candidate biomarkers and persistent metabolic abnormalities.

## Introduction

Hyperthyroidism is the most commonly diagnosed endocrinopathy in cats [1]. Most cases are caused by benign adenomatous thyroid tissue. A small proportion of patients (approximately 2–3%) are due to thyroid carcinoma [2, 3]. The underlying etiology of hyperthyroidism in cats has yet to be identified; however, published studies suggest the cause of feline hyperthyroidism to be multifactorial, with dietary and environmental risk factors likely contributing [1, 4–6]. Autonomous secretion of thyroxine and triiodothyronine (T_4_ and T_3_, respectively) are responsible for the disease’s clinical and metabolic manifestations.

Clinical abnormalities associated with hyperthyroidism in cats include muscle catabolism, generalized weakness, polyphagia, polyuria and polydipsia, behavioral changes, hair coat changes, cardiomyopathy, hypertension, and vomiting/diarrhea. These sequelae are detrimental to hyperthyroid cats’ health and quality of life. Hyperthyroid cats often have alterations in serum biochemical markers, the most common of which is an increased alanine transferase, reflecting hepatocellular injury or enzyme induction [7]. It is posited that increased metabolic demand and oxidative stress drive increases in this enzyme.

Metabolomic profiles in human hyperthyroid patients (both naturally occurring and experimentally induced) demonstrate persistent metabolic alterations despite achieving euthyroid status in some cases [8]. Persistent alterations in the metabolome after successful radioactive iodine I-131 treatment in cats would be attractive targets for developing novel therapeutic interventions.

**Fig 1:**
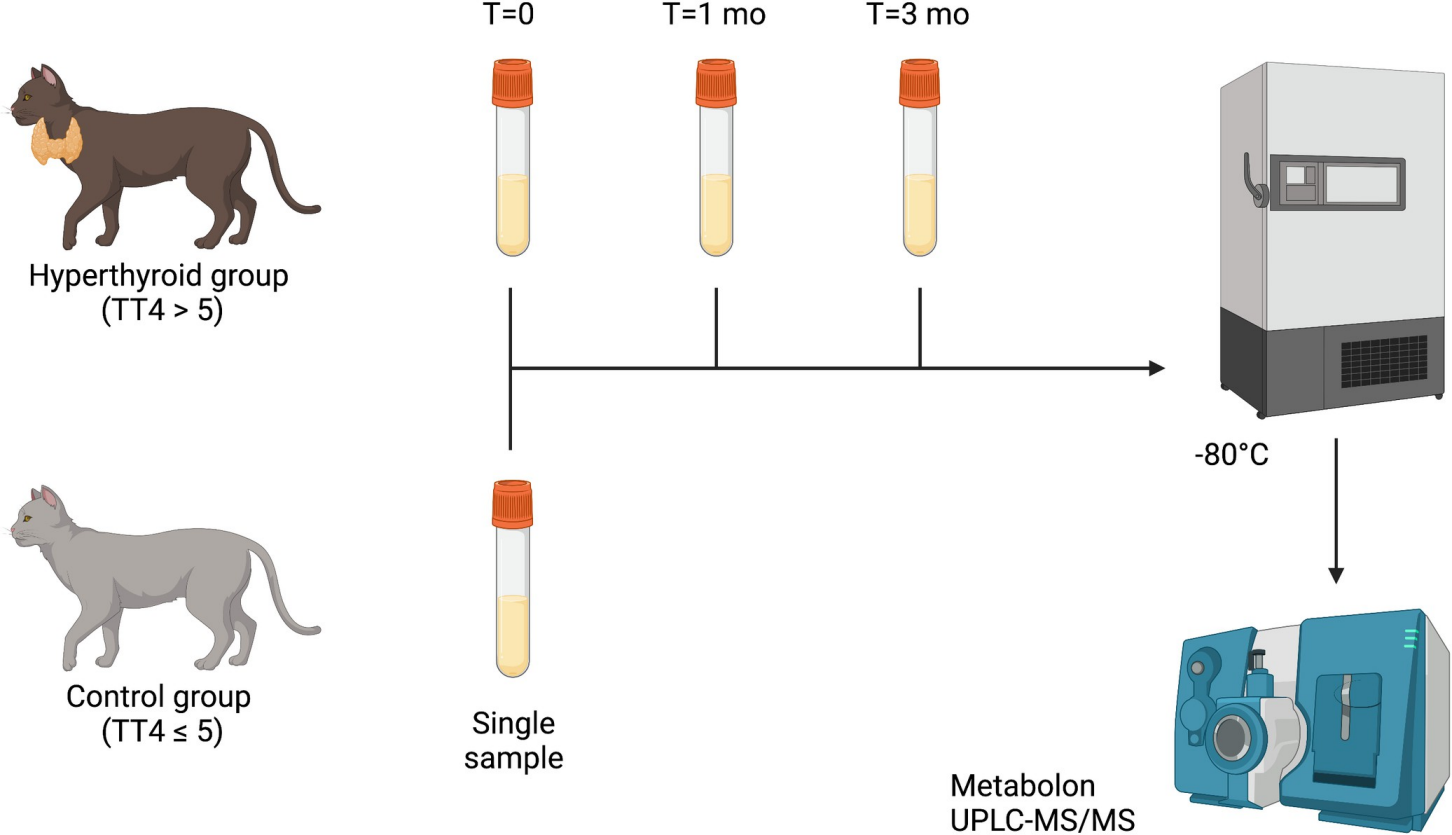
Methodology overview. Serum was collected from hyperthyroid cats (n = 7) before (T = 0) and after (at T = 1 mo and T = 3 mo) I131 treatment. A single sample was collected from control cats (n = 12). Samples were stored for subsequent untargeted metabolic profiling and analysis. Created with Biorender.com.

We hypothesized that hyperthyroid cats would possess distinct metabolomic signatures and that some metabolomic changes would persist after (I-131) treatment. Additionally, an untargeted metabolomics approach has the potential to identify candidate biomarkers that may aid in diagnosing challenging hyperthyroid cases. Therefore, we leveraged an untargeted metabolomic approach (Fig 1) to (i) define the metabolomic “fingerprint” of hyperthyroid cats, (ii) identify critical metabolic pathways affected in feline hyperthyroidism, and (iii) identify persistent alterations in metabolomic profiles in cats following I-131 treatment and achievement of euthyroid state.

## Results and discussion

### Animals

Ninety-six cats were initially eligible for enrollment for I-131 treatment between September 14, 2020, and April 29, 2022. However, six cats did not undergo treatment for various reasons, resulting in 90 eligible cats. Twelve cats were enrolled with informed client consent. All other eligible cats were not enrolled due to declined client consent (most cases), a decision not to treat with I-131, or both. Serum samples were collected before I-131 treatment (HYP-T0), at one (HYP-T1), and at three (HYP-T3) months post-treatment (Fig 1). Follow-up timepoints were chosen based on typical evaluation times post-I-131 treatment. All cats treated cats in the study were euthyroid at both follow-up time points. Five cats were excluded from the study because a complete set of samples was not obtained due to loss to follow-up (n = 4) or patient mortality (n = 1). Seven hyperthyroid cats (n = 3 male, n = 4 female) completed the study time points and were included in the final analysis. The control group comprised 12 cats. Age, weight, and body condition scores were similar between hyperthyroid and control cats (Table 1).

**Table 1:** Demographic and clinical characteristics of hyperthyroid and healthy control cats included in this study.

	Hyperthyroid (Baseline)		Control		
	Median	Range	Median	Range	P value
Age (years)	12	9-14	14.5	11-16.5	0.1525
Weight (kg)	3.76	3.4-4.4	4.65	3-6.35	0.1375
BCS (1-9 scale)	5	4-5	4.5	3-8	0.7932
T4 (μg/dL, RR = 2-5)	16.6	8-29.4	3.395	1.43-4.34	<0.0001

BCS = Body condition score. T4 = Total serum thyroxine.

### Serum metabolites

**Fig 2:**
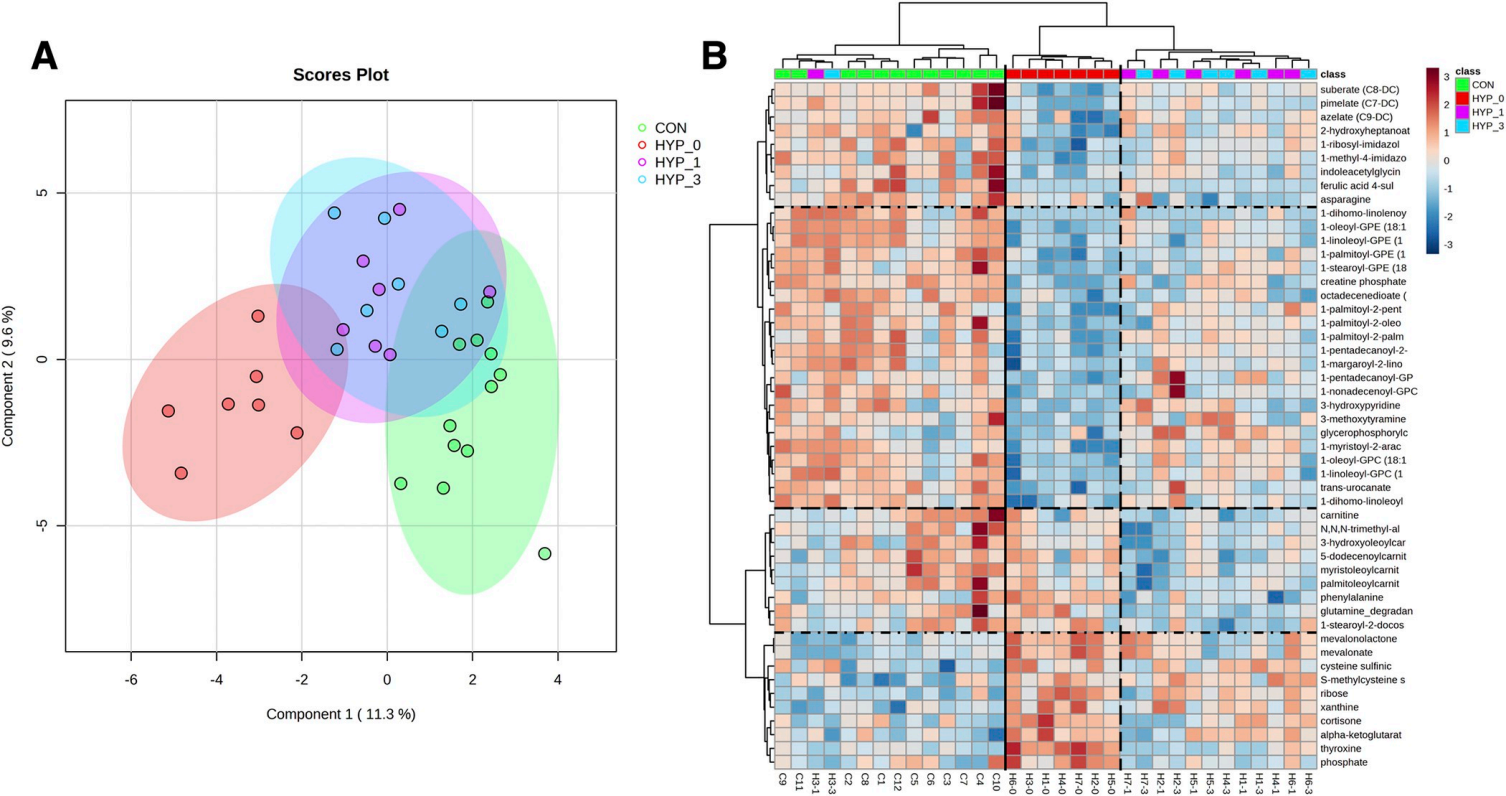
Cluster analysis of feline metabolome data. (A) Sparse Partial Least Squares—Discriminant Analysis (sPLS-DA) plot. Dots indicate individual cats, and shading highlights clustering. (B) Heatmap of 25 metabolites with the smallest p values, segregated into clades based on patterns of relative magnitude. Colors indicating groups are provided in legends. CON = healthy control cats, HYP-T0 = hyperthyroid time 0, HYP-T1 = hyperthyroid cats 1-month post-I131 treatment, and HYP-T3 = hyperthyroid cats 3 months post-I131 treatment.

Hyperthyroid cats sampled before I-131 treatment (HYP-T0) had a distinct metabolic signature (S1 Table) with 277 metabolites significantly different than controls (70 increased, 207 decreased). Data reduction analysis (Fig 2A) revealed separate clustering of hyperthyroid (HYP-T0) and CON cats with intermediate phenotypes after treatment (HYP-T1 & HYP-T3). Cluster analysis demonstrated a similar pattern with clades segregating hyperthyroid cats before I-131, after treatment, and control cats separately, except for one hyperthyroid cat that segregated with controls after treatment (Fig 2B).

### Metabolic pathways

**Fig 3:**
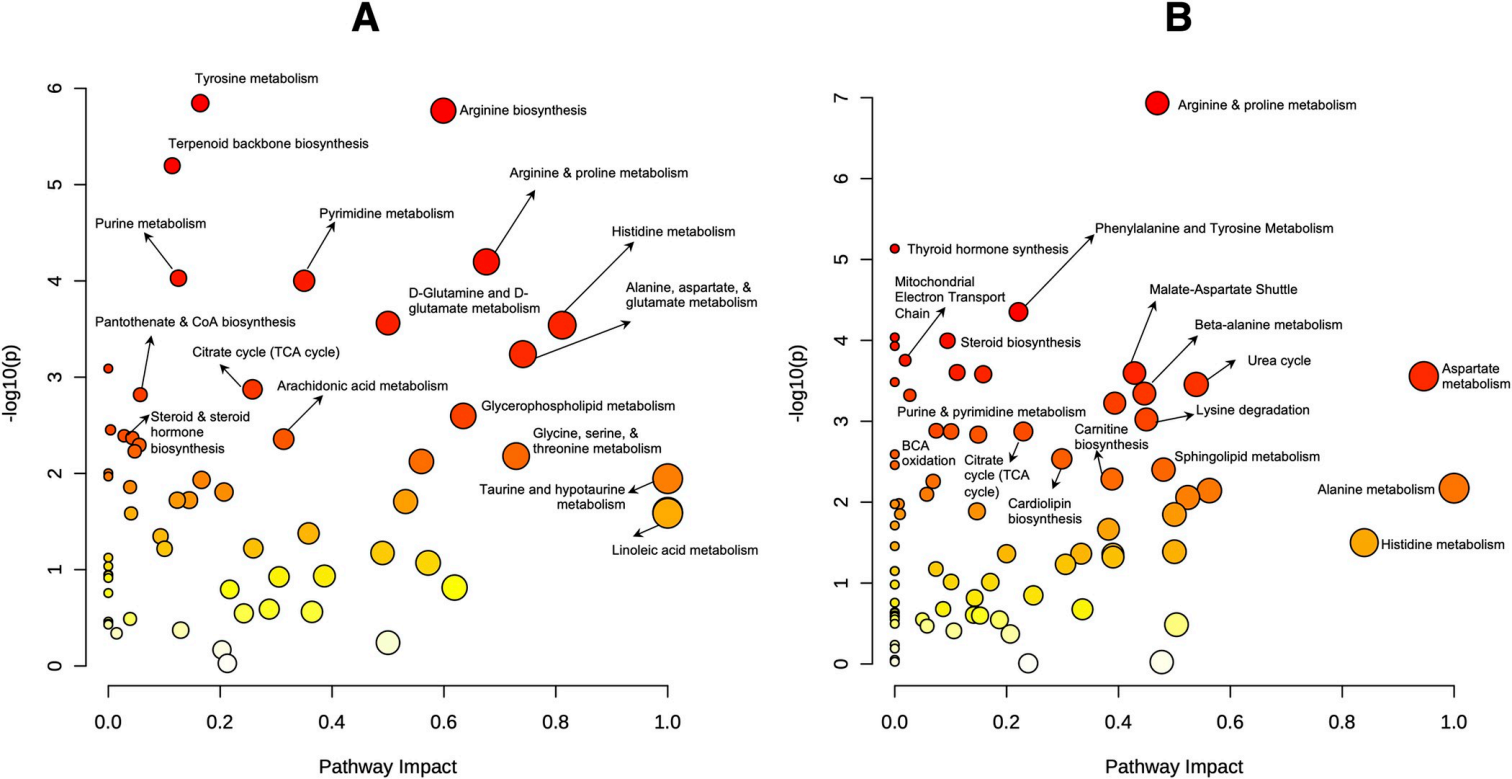
Pathway analysis. Topology maps of affected Kyoto Encyclopedia of Genes and Genomes (A) and Small Molecule Pathway Database (B) pathways impacted in serum metabolomes of hyperthyroid cats (n = 7) versus healthy control cats (n = 12). Highly significant and impacted pathways are annotated. Each circle represents a pathway, circle sizes correspond to pathway impact, and the color gradient corresponds to pathway significance.

The metabolic signatures of hyperthyroid cats (HYP-T0) indicated alterations in several metabolic pathways (S1 Fig). The pathway most impacted in hyperthyroid cats was taurine and hypotaurine metabolism (Fig 3 and S2 Table). After taurine and hypotaurine metabolism, amino acid metabolic and biosynthetic pathways were the most impacted pathways (Fig 3 and S2 Table). Other notable pathways included the TCA cycle, glycerophospholipid metabolism, arachidonic acid metabolism, nucleotide metabolism, steroid and steroid hormone (cortisol) biosynthesis, carnitine biosynthesis, and B vitamin metabolism (S2 Table). These findings align with changes observed in hyperthyroid humans, notably altered fatty acid biosynthesis and metabolism [9], indicating potential translational relevance.

### Metabolites defining feline hyperthyroidism

**Fig 4:**
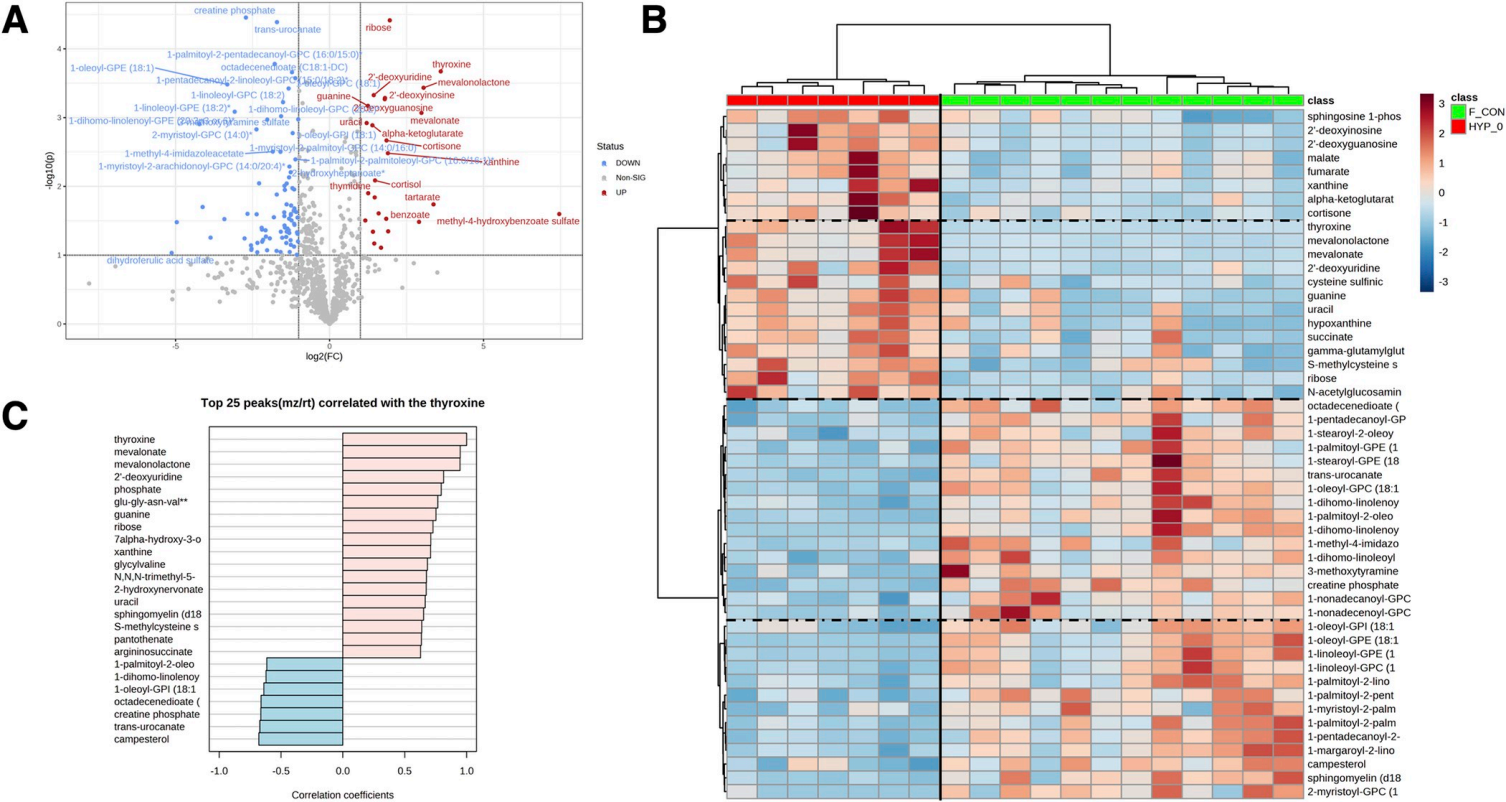
Comparison of healthy controls to hyperthyroid state. (A) Volcano plots of significant metabolites in hyperthyroid cats (n = 7) versus healthy control cats (n = 12). Metabolites significantly (*P* < 0.05) increased (red) or decreased (blue) greater than 2-fold are depicted by single dots. (B) Heatmap of 25 metabolites with the smallest *P* values, segregated into clades based on patterns of relative magnitude. Colors indicating groups are provided in legends. CON = healthy control cats, HYP-T0 = hyperthyroid time 0. (C) Correlation plot of metabolites best correlated to thyroxine.

Several metabolites were significantly increased or decreased greater than two-fold in hyperthyroid (HYP-T0) cats compared to healthy controls (Fig 4A), with a larger proportion of metabolites being decreased. When comparing cats in the hyperthyroid state (HYP-T0) to euthyroid control cats, cluster analysis segregated the groups into two distinct clades (Fig 4B). Notably, ribose, cortisol, alpha-ketoglutarate, and mevalonate increased. While creatine phosphate, transurocanate, and several lipid metabolites were decreased. Many of these metabolites also correlated with thyroxine (Fig 4C). Studies in hyperthyroid humans have demonstrated increased alpha-ketoglutarate and creatine [8, 10]. Decreased creatine phosphate in hyperthyroid cats and increased creatine in hyperthyroid humans may reflect species differences or could imply altered conversion. Lower creatine phosphate (phosphocreatine) may reflect lower muscle masses often seen in hyperthyroid cats [11, 12]. As creatine phosphate serves as a rapidly mobilizable reserve of high-energy phosphates in skeletal muscle, myocardium, and the brain, reduced serum levels also reflect changes in energy metabolism at these sites [13, 14]. In this study, post-I-131 treatment creatine phosphate levels improved but were still significantly reduced compared to controls, further indicating persistent negative energy balance in previously hyperthyroid cats. Increased alpha-ketoglutarate may be consistent with an overall increase in TCA cycle activity associated with a hypermetabolic state [15]. Although these levels decreased post-I-131, they were still slightly elevated compared to control cats, indicating a persistent hypermetabolic state during our observation period.

**Fig 5:**
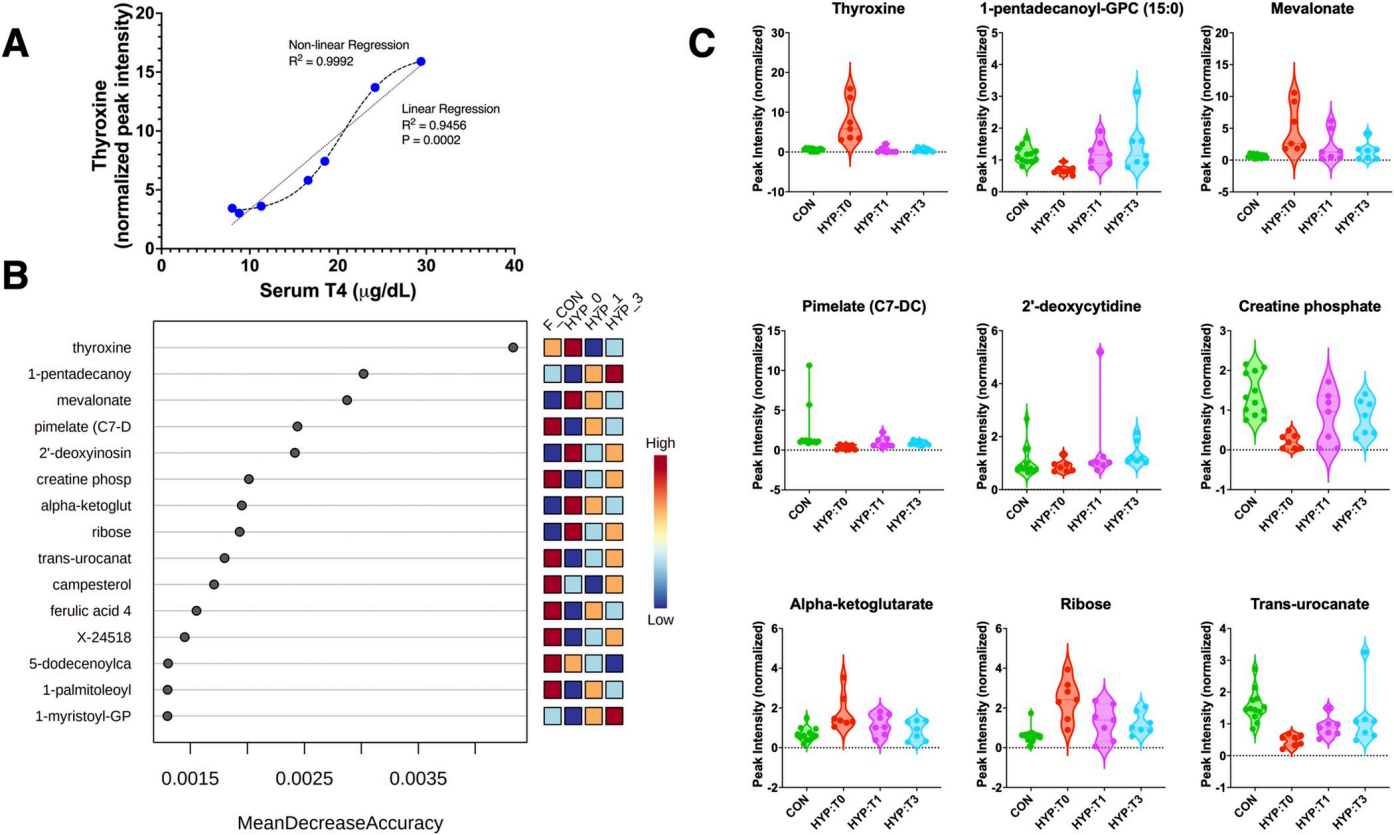
Model analysis in hyperthyroid cats. (A) Comparison of total T4 values (measured by radioimmunoassay) vs. thyroxine values obtained through metabolomic profiling. (B) Random Forest Analysis variation importance plots for serum metabolites are depicted. (C) Individual plots of the top nine metabolites identified by random forest analysis. CON = healthy control cats, HYP-T0 = hyperthyroid time 0, HYP-T1 = hyperthyroid cats 1-month post-I131 treatment, and HYP-T3 = hyperthyroid cats 3 months post-I131 treatment.

We conducted random forest analysis (RFA) to identify the metabolites that best classified groups. When all groups were included in the model, thyroxine was the best distinguishing metabolite for the model, in accord with diagnostic standards where serum total T_4_ is the screening test of choice for diagnosing hyperthyroidism in cats [16–19]. The accuracy of the mass spectrometry measured thyroxine was compared to total T4 by radioimmunoassay, and agreement by linear and non-linear regression was excellent (Fig 5A). After thyroxine, the best model discriminators were 1-pentadecanoyl-GPC and mevalonate (Fig 5B and 5C).

**Fig 6:**
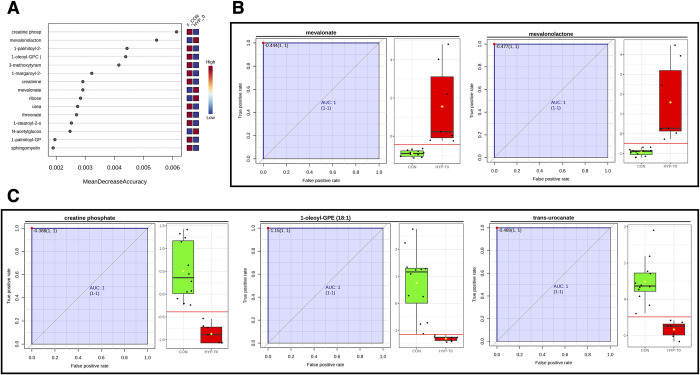
Features distinguishing cats in a hyperthyroid state from healthy cats. (A) Random forest analysis was conducted on metabolomic results from hyperthyroid cats before treatment (HYP-T0) and healthy controls. Univariate biomarker analysis conducted by receiver-operator characteristics identified robust novel candidate biomarkers. Mevalonate and mevalonolactone were higher (A), and creatine phosphate, 1-oleoyl-GPE (18:1), and trans-urocanate (B) were lower in hyperthyroid cats.

We conducted RFA and univariate biomarker analysis (receiver-operator characteristics, ROC), comparing controls to HYP-T0. Three metabolites were shared with the RFA model, including post-treatment data, creatine phosphate, mevalonate, and ribose (Fig 6A). In this RFA model, mevalonolactone was the second-best metabolite. The out-of-bag error rate was 0.105, with class errors of 0.0833 and 0.143 for hyperthyroid and control cats, respectively. Complementing the RFA results, we identified five metabolites by ROC with area under the curves of 1.0, indicating excellent discrimination between healthy control and hyperthyroid (HYP-T0) cats. Two metabolites, mevalonate and mevalonolactone, were increased (Fig 6B). In comparison, three metabolites, creatine phosphate, 1oleol-GPE (18:1), and transurocanate, were decreased in hyperthyroid cats (Fig 6C). Key components of the mevalonate pathway, the hydroxy fatty acids mevalonate and mevalonolactone, are essential for cholesterol biosynthesis. The enzyme 3-hydroxy-3-methylglutaryl-coenzyme A (HMG-CoA) reductase synthesizes these lipids and is the rate-limiting step in cholesterol biosynthesis [20]. Thus, our findings further highlight similarly altered cholesterol and steroid hormonogenesis observed in hyperthyroid humans [21–23].

Similarly, transurocanate was reduced in the hyperthyroid state and did not return to normal levels post-I-131 treatment, further suggesting persistent alterations in the TCA cycle. Ribose was elevated in hyperthyroid cats compared to controls but did not return to normal following I-131 treatment and euthyroid state. As a vital component of DNA, RNA, ATP, ADP, and AMP, this supports altered carbohydrate and energy metabolism in hyperthyroid cats, corroborated by differences in fructosamine levels between healthy and hyperthyroid cats [24].

Our study aimed to find new biomarkers for feline hyperthyroidism, especially in challenging cases. With routine screening becoming more common, cats are diagnosed earlier, sometimes with milder symptoms [25, 26]. This could be why 10% of hyperthyroid cats, and up to 30% with early or mild cases, show normal serum total T4 levels [16, 17, 19]. These cases are often linked to early-stage hyperthyroidism or concurrent non-thyroidal illness [16]. Tests like free T4, T3 suppression, and thyrotropin-releasing hormone stimulation are suggested but can be unreliable with non-thyroidal illness and challenging to perform [19, 27]. Another approach is retesting serum T4 levels or starting treatment with methimazole and monitoring the response [27]. Given these challenges, new biomarkers that differentiate euthyroid from hyperthyroid cats with non-thyroidal illness or early hyperthyroidism would be useful. Further evaluation of biomarkers like mevalonate/mevalonolactone and creatine phosphate is needed, though they may not fully differentiate occult hyperthyroidism as they correlate with thyroxine levels in our study.

### Metabolites of additional clinical interest

**Fig 7:**
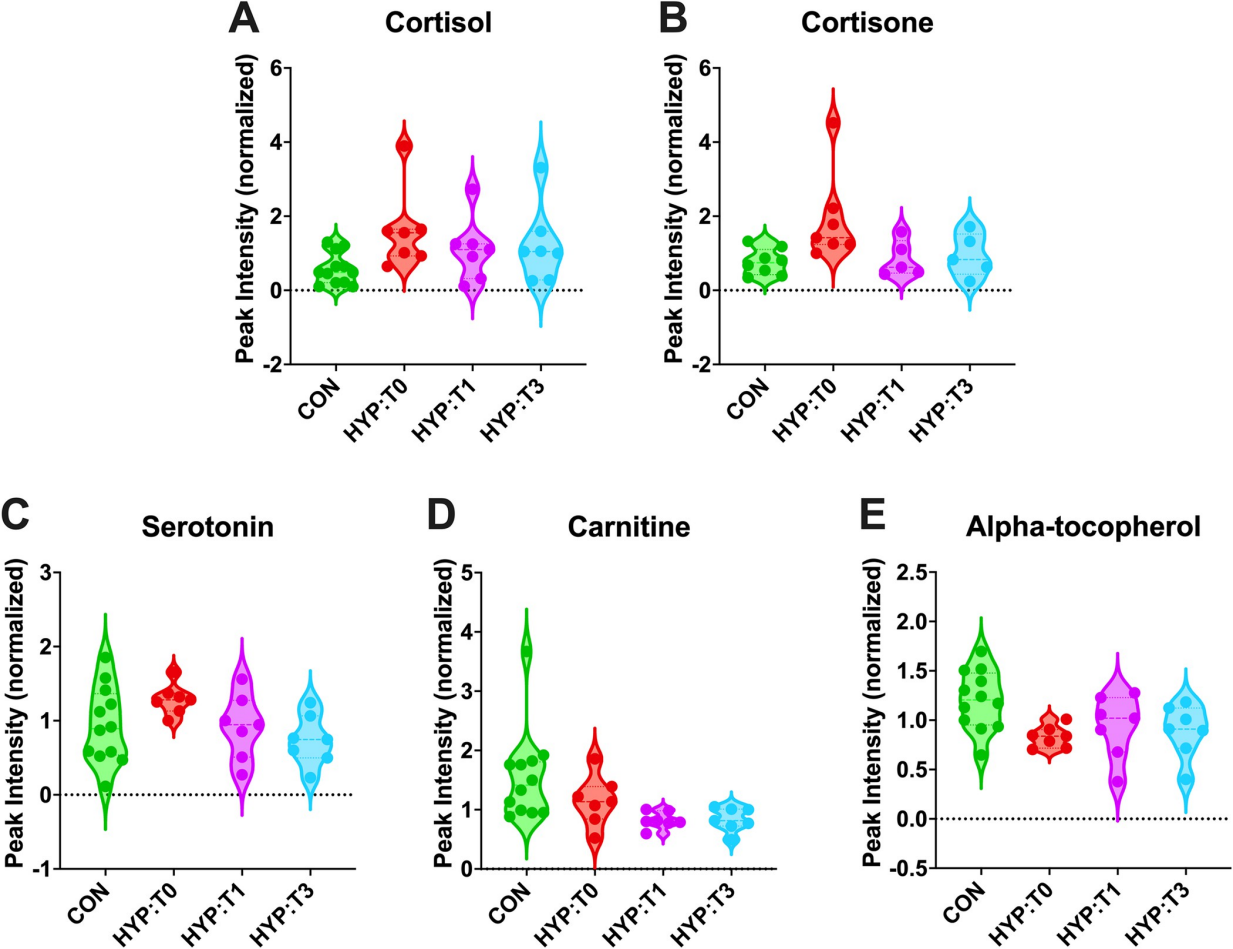
Selected metabolites of clinical interest. Violin plots of (A) cortisol, (B) cortisone, (C) serotonin, (D) carnitine, and (E) alpha-tocopherol. Dots indicate individual cat values. CON = healthy control cats, HYP:T0 = hyperthyroid time 0, HYP:T1 = hyperthyroid cats 1-month post-I131 treatment, and HYP:T3 = hyperthyroid cats 3 months post-I131 treatment.

Several metabolites were notable due to their clinical implications, persistent alteration after treatment, or both. Hyperthyroid cats (HYP-T0) had significantly higher cortisol and cortisone levels (Fig 7A and 7B). Changes in cortisol and cortisone combined with the impacts on lipid pathways prompted us to compare cholesterol concentrations from metabolomic and biochemistry analysis. Although cortisol was in the reference range for all hyperthyroid cats in the study, hyperthyroid cats had significantly lower (P < 0.02) cholesterol concentrations measured by biochemistry and metabolomic analysis (S2 Fig and S1 Table).

While hypocholesterolemia isn’t commonly noted in hyperthyroid cats, our observations indicate that cholesterol levels in these cats often trend toward the lower end of the reference range. Furthermore, a study reported a general increase in serum cholesterol concentrations in treated hyperthyroid cats [28]. Although our data align with the lower cholesterol trend in hyperthyroid cats, levels typically remain within diagnostic laboratory reference ranges. In humans, hyperthyroidism leads to increased cholesterol excretion and turnover of low-density lipoproteins, resulting in decreased total and low-density lipoproteins. Conversely, high-density lipoproteins are either reduced or unaffected [29]. Cholesterol levels normalize in humans once a euthyroid state is achieved, indicating normalization of cholesterol metabolism [10, 30]. However, we did not observe this phenomenon in our cohort of cats post-I-131 treatment, suggesting cholesterol homeostasis may take longer to normalize in cats. Similarly, despite achieving a euthyroid state following I-131 therapy, higher mevalonate levels in hyperthyroid cats did not return to levels comparable to euthyroid controls.

Hypercortisolemia has previously been documented in hyperthyroid cats, which was corroborated by our results [31, 32]. However, to our knowledge, blood cortisol levels following hyperthyroidism treatment in cats have yet to be studied. Our data reveal persistent hypercortisolemia even after achieving euthyroid status with I-131 therapy.

In one study, hyperthyroid cats had significantly higher cortisol concentrations post-adrenocorticotropic hormone stimulation; however, adrenal gland size and urine cortisol:creatinine ratios did not differ significantly from controls [31]. When urine cortisol:creatinine ratios were measured following I-131 therapy, those ratios were significantly reduced compared to baseline in the hyperthyroid cats [32]. Despite expectations of cortisol normalization post-treatment, our findings indicate persistent alterations in steroid biosynthetic pathways for at least three months, supported by increased adrenal size measured by ultrasound in treated hyperthyroid cats [33].

Median serotonin levels, a neurotransmitter that could contribute to some clinical signs of hyperthyroidism, were higher in hyperthyroid cats (Fig 7C). Serum levels of two metabolites of nutritional relevance, carnitine and alpha-tocopherol, were lower (T0 vs. CON), persisting after I-131 treatment (Fig 7D and 7E). Additionally, 66 (T1 vs. CON) and 64 (T3 vs. CON) metabolite differences persisted after treatment. Although serotonin levels showed some overlap between hyperthyroid and healthy cats, overall concentrations were higher in hyperthyroid cats. To the authors’ knowledge, serotonin has not been evaluated in hyperthyroid cats but has been shown to be increased in rodent models of hyperthyroidism [34, 35]. This potential increase in serotonin may partly explain clinical manifestations in hyperthyroid cats, including behavioral changes, vomiting, and diarrhea as these signs are seen in serotonin syndrome [36]. Elevated serotonin and cortisol levels may also contribute to the variability in clinical signs among individual hyperthyroid cats.

We identified two potential nutritional targets for achieving euthyroidism in cats post-I-131 treatment: vitamin E and carnitine. α-Tocopherol, the most bioactive form of vitamin E, acts as a crucial antioxidant, shielding membranes from oxidative damage by peroxyl radicals. Reduced α-tocopherol levels have been noted in hyperthyroid people [37], with studies in rodent models and humans indicating benefits from vitamin E supplementation in managing hyperthyroidism [38, 39]. Cats, having a less favorable glutathione status than dogs, are particularly susceptible to oxidative injury, further suggesting potential benefits from vitamin E supplementation [40]. However, one study found no disparities in vitamin E concentrations between control and hyperthyroid cats pre- and post-I-131 treatment [39], necessitating further investigation into vitamin E status in hyperthyroid cats and any benefit for its supplementation.

Carnitine, essential for β-oxidation, facilitates the transport of long-chain fatty acids into mitochondria for energy production [41]. Given its metabolic role, carnitine is concentrated in skeletal and cardiac muscle and other tissues that metabolize fatty acids as an energy source [42]. Post-I-131 treatment, significantly lower carnitine levels could perpetuate altered energy metabolism, inefficient lipid metabolism, and impaired β-oxidation in achieving euthyroid status. Therefore, carnitine supplementation may prove beneficial in restoring metabolic balance in euthyroid cats following I-131 therapy.

Hyperthyroidism results in a hypermetabolic state, leading to altered lipid, protein, and carbohydrate metabolism with increased resting energy requirements [43–45]. This hypermetabolic state is presumed to be reversed once the euthyroid state is achieved. Our study revealed a severe, global alteration in the metabolome of hyperthyroid cats. Although the metabolome trended towards that of the euthyroid control group post-treatment, certain metabolites in lipid, protein, and carbohydrate metabolic pathways remained persistently altered despite achieving euthyroid status, consistent with findings in human hyperthyroidism [10, 46–48].

This study’s main limitation was a modest sample size obtained from a single clinical site. The sample size was targeted based on previous metabolomic studies conducted in cats and dogs and guidelines from the metabolomic lab that conducted the analysis. Further supporting the validity of our results is the robust metabolic phenotype occurring in hyperthyroidism. Future studies with multiple sites may be interesting in evaluating geographical differences in hyperthyroid cat metabolomes. This approach might also reveal candidate location-related risk factors. Our follow-up period was relatively short. Therefore, it is possible that a longer monitoring period would disclose the normalization of more metabolic perturbations. A longer longitudinal study would be needed to determine if supplementing any of the targeted metabolic disturbances described above is (i) beneficial and (ii) required long or short-term. Finally, the nature of untargeted metabolomic analysis only provides a semi-quantitative measurement of analytes. Therefore, it is imperative to conduct follow-up studies that quantitatively measure metabolites of interest. These studies should ideally include training and validation steps in data analysis to validate findings from untargeted metabolomic studies like ours.

Our study defined the metabolome of hyperthyroid cats, which demonstrates significant alterations in lipid, carbohydrate, and protein metabolism. Some of these metabolomic changes persisted after I-131 treatment despite a return to euthyroidism. These persistent changes were associated with oxidative stress, cholesterol and steroid hormonogenesis, energy metabolism, and lipid metabolism. These data suggest that recovery to normal serum T_4_ concentrations following I-131 therapy may belie persistent metabolic derangements in successfully treated hyperthyroid cats. Further studies are needed to determine if hyperthyroid cats could benefit from additional nutritional or medical treatments to address these lasting metabolomic derangements.

## Materials and methods

### Animals

We conducted this case-control study from 9/14/2020 to 4/29/2022. This study was approved by the Cornell University Institutional Animal Care and Use Committee for the use of vertebrates in research (#2014–0052). All experiments were conducted following all applicable guidelines and regulations and are reported to be consistent with ARRIVE guidelines. Informed consent was obtained from all owners before enrollment of any case or control cat.

Hyperthyroid case cats (HYP) were enrolled from client-owned patients presented to Cornell University Hospital for Animals (CUHA) Internal Medicine Service for I-131 treatment. Cats with mild kidney disease (International Renal Interest Society Stage 1 or 2), chronic enteropathy, or cardiac abnormalities attributable to hyperthyroidism were permitted. Patients demonstrating radiopharmaceutical uptake consistent with neoplasia were excluded from the study. Any prior treatment, such as methimazole, was discontinued for at least ten days before receiving I-131 therapy. The dosage of I-131 was selected based on each patient’s serum T4 level and thyroid scintigraphy results. Doses of I-131 were administered based on our hospital’s standard protocol of 2 mCi for T4 of 5–10 ug/dL, 4 mCi for T4 >10–20 ug/dL, and 4 or 6 mCi at the clinician’s discretion for T4 > 20 ug/dL that was developed through a previous study [49].

Privately owned control (CON) cats were recruited from staff and students at the Cornell University College of Veterinary Medicine, targeting a demographic population similar to the one we obtained for the HYP group. A normal serum T4 value was required for CON group inclusion. All other inclusion and exclusion criteria for the HYP group were also applied to the CON group.

For both case and control cats, a complete blood count, serum chemistry profile, total T4, and urinalysis were performed (by the New York State Veterinary Diagnostic Laboratory) as part of routine diagnostic testing. Additionally, these parameters were rechecked in case cats, and serum was collected for metabolomic analysis at 1 month and 3 months following I131 therapy (Fig 1).

### Sample collection and processing

Fasted blood samples were acquired by venipuncture of peripheral veins. The phlebotomist chose the site at their discretion. In most cases, blood was collected contemporaneously with diagnostic blood sampling. Whole blood was collected into 4 ml additive-free tubes (BD Vacutainer^®^). After clotting (~15 minutes), tubes were centrifuged at 676 x g for 10 minutes at room temperature to yield serum. Serum was collected with transfer pipettes and stored in 2 ml plastic tubes. All serum samples were stored at -80°C and shipped on dry ice for analysis.

### Metabolomic profiling

Metabolon (Metabolon, Inc, 617 Davis Drive, Suite 100, Morrisville, NC 27560) conducted metabolomic profiling on serum samples as previously described [50–53]. Samples were prepared using the automated MicroLab STAR^®^ system from Hamilton Company. Proteins were precipitated with methanol under vigorous shaking for 2 min (Glen Mills GenoGrinder 2000), followed by centrifugation to remove protein, dissociate small molecules bound to protein or trapped in the precipitated protein matrix, and recover chemically diverse metabolites. The resulting extract was divided into five fractions: two for analysis by two separate reverse phases (RP)/UPLC-MS/MS methods with positive ion mode electrospray ionization (ESI), one for analysis by RP/UPLC-MS/MS with negative ion mode ESI, one for analysis by HILIC/UPLC-MS/MS with negative ion mode ESI, and one sample was reserved for backup. Samples were placed briefly on a TurboVap^®^ (Zymark) to remove the organic solvent. The sample extracts were stored overnight under nitrogen before preparation for analysis.

All methods utilized a Waters ACQUITY ultra-performance liquid chromatography (UPLC) and a Thermo Scientific Q-Exactive high resolution/accurate mass spectrometer interfaced with a heated electrospray ionization (HESI-II) source and Orbitrap mass analyzer operated at 35,000 mass resolution.

The informatics system consisted of four major components, the Laboratory Information Management System (LIMS), the data extraction and peak-identification software, data processing tools for QC and compound identification, and a collection of information interpretation and visualization tools for data analysts. These informatics components’ hardware and software foundations were the LAN backbone and a database server running Oracle 10.2.0.1 Enterprise Edition.

Metabolon maintains a library based on authenticated standards that contains the retention time/index (RI), mass-to-charge ratio (m/z), and chromatographic data (including MS/MS spectral data) on all molecules present in the library. Furthermore, biochemical identifications are based on three criteria: retention index within a narrow RI window of the proposed identification, accurate mass match to the library +/- 10 ppm, and the MS/MS forward and reverse scores between the experimental data and authentic standards. The MS/MS scores are based on comparing the ions present in the observed spectrum to those in the library spectrum. While there may be similarities between these molecules based on one of these factors, all three data points can be used to distinguish and differentiate biochemicals.

The Human Metabolome Database (https://hmdb.ca/) was searched for reference information on selected metabolites.

### Statistical analysis

A minimum of 7–10 cases was targeted based on Metabolon’s sample size guidelines. Descriptive statistics are reported as medians and ranges. Due to the sample size, we did not conduct normality testing and applied non-parametric statistics. We compared age, weight, body condition score, and total T4 by the Mann-Whitney test using commercial software (Prism 9.0 or later, GraphPad, San Diego, CA 92108, RRID:SCR_002798).

Metabolon’s initial standard statistical analyses are performed in ArrayStudio/Jupyter Notebook on log-transformed data. For those analyses not standard in ArrayStudio/Jupyter Notebook, the programs R (http://cran.r-project.org/) or JMP are used. The Welch’s two-sample *t*-test compared control (CON) cats to hyperthyroid (HYP) cats at each time point (i.e., CON:HYP-T0, CON:HYP-T1, and CON:HYP-T3). The Matched Pair t-test compared hyperthyroid cats between time points (i.e., HYP-T0: HYP-T-1, HYP-T0:HYP-T3, and HYP-T1:HYP-T3).

Additional statistical analyses and data visualizations (including volcano plots, random forest analysis, and biomarker analysis) were performed with MetaboAnalyst 5.0 (www.metaboanalyst.ca, RRID:SCR_015539). Normalized data (S1 File) were log-transformed, and Pareto data scaling was applied. Fold-change and T-tests further identified significantly increased or decreased metabolites. The sparse partial least squares—discriminant analysis (sPLS-DA) method was chosen for data reduction analysis [54]. Pathway analysis was conducted using the human Kyoto Encyclopedia of Genes and Genomes (KEGG) and Small Molecule Pathway Database databases. A P value of < 0.05 established significance.

## Supporting information

S1 File Normalized peak intensity metabolomic data used for analyses. (CSV)

S1 Fig Detailed pathway map of significant metabolites between hyperthyroid (n = 7) versus control (n = 12) cats. Circle size indicates magnitude of difference (increased in red, decreased in blue) between metabolites in hyperthyroid cats vs. controls.
(PDF)

S2 Fig Serum cholesterol concentrations in hyperthyroid cats and euthyroid controls. (A) comparison of cholesterol concentrations measured in euthyroid control cats compared to hyperthyroid cats before treatment. (B) Changes in serum cholesterol measured in hyperthyroid cats before (HT0), 1 month (HT1) and 3 months (HT3) after I-131 treatment. Lines connect the same cat measured serially. *P < 0.05.
(TIF)

S1 Table Results of significant metabolites in hyperthyroid (n = 7) versus control (n = 12) cats. (XLSX)

S2 Table Top 25 metabolic pathways affected in hyperthyroid (n = 7) versus control (n = 12) cats. (XLSX)
