# Supplementary material for: Serum metabolome analysis in hyperthyroid cats before and after radioactive iodine therapy
Source: PLoS One. 2024 Jun 10;19(6):e0305271. doi: 10.1371/journal.pone.0305271 (PMC11164369; doi:10.1371/journal.pone.0305271)
Supplement: S1 Fig — Circle size indicates magnitude of difference (increased in red, decreased in blue) between metabolites in hyperthyroid cats vs. controls. (PDF) [file pone.0305271.s002.pdf]

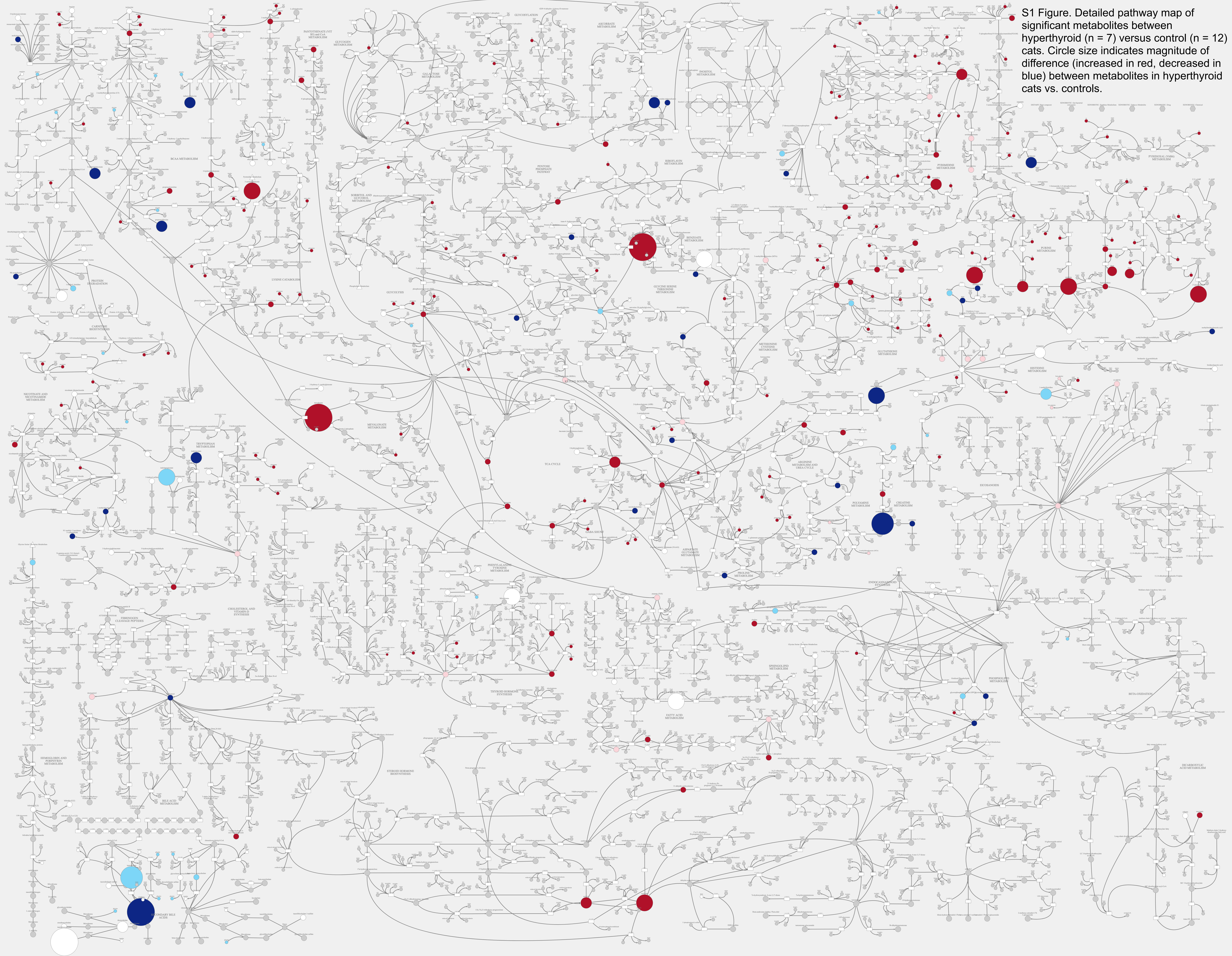

S1 Figure. Detailed pathway map of significant metabolites between hyperthyroid (n = 7) versus control (n = 12) cats. Circle size indicates magnitude of difference (increased in red, decreased in blue) between metabolites in hyperthyroid cats vs. controls.
