# Supplementary figures and images for: Serum metabolome analysis in hyperthyroid cats before and after radioactive iodine therapy
Source: PLoS One. 2024 Jun 10;19(6):e0305271. doi: 10.1371/journal.pone.0305271 (PMC11164369; doi:10.1371/journal.pone.0305271)

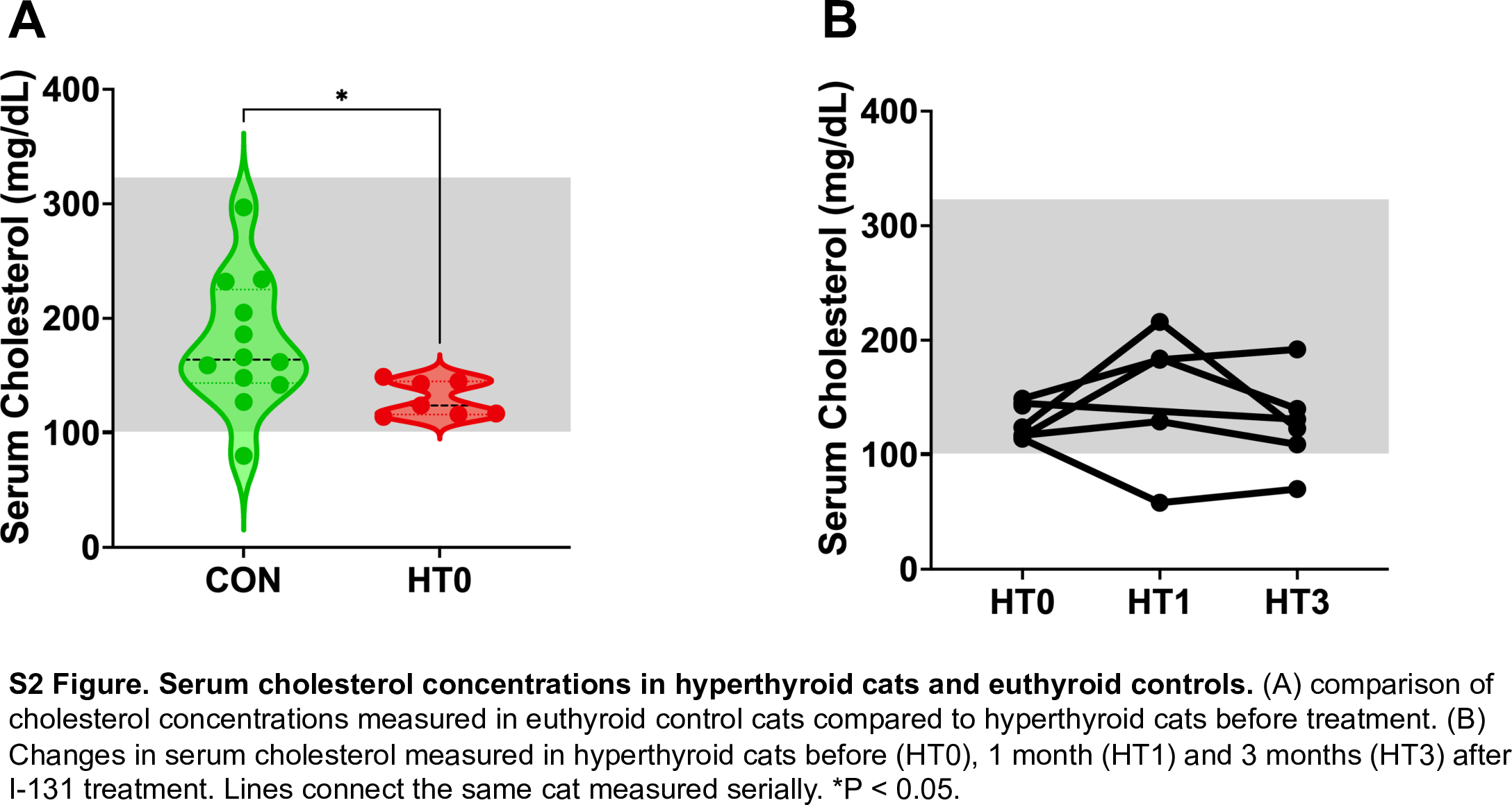

Supplement: S2 Fig — (A) comparison of cholesterol concentrations measured in euthyroid control cats compared to hyperthyroid cats before treatment. (B) Changes in serum cholesterol measured in hyperthyroid cats before (HT0), 1 month (HT1) and 3 months (HT3) after I-131 treatment. Lines connect the same cat measured serially. *P < 0.05. (TIF) [file pone.0305271.s003.tif]
